# Prenatal Air Pollution Exposure and Placental DNA Methylation Changes: Implications on Fetal Development and Future Disease Susceptibility

**DOI:** 10.3390/cells10113025

**Published:** 2021-11-05

**Authors:** Terisha Ghazi, Pragalathan Naidoo, Rajen N. Naidoo, Anil A. Chuturgoon

**Affiliations:** 1 Discipline of Medical Biochemistry, School of Laboratory Medicine and Medical Sciences, College of Health Sciences, University of KwaZulu-Natal, Durban 4041, South Africa; GhaziT@ukzn.ac.za (T.G.); naidoop5@ukzn.ac.za (P.N.); 2 Discipline of Occupational and Environmental Health, School of Nursing and Public Health, College of Health Sciences, University of KwaZulu-Natal, Durban 4041, South Africa; naidoon@ukzn.ac.za

**Keywords:** DOHaD concept, air pollution, heavy metals, pregnancy, placenta, DNA methylation

## Abstract

The Developmental Origins of Health and Disease (DOHaD) concept postulates that in utero exposures influence fetal programming and health in later life. Throughout pregnancy, the placenta plays a central role in fetal programming; it regulates the in utero environment and acts as a gatekeeper for nutrient and waste exchange between the mother and the fetus. Maternal exposure to air pollution, including heavy metals, can reach the placenta, where they alter DNA methylation patterns, leading to changes in placental function and fetal reprogramming. This review explores the current knowledge on placental DNA methylation changes associated with prenatal air pollution (including heavy metals) exposure and highlights its effects on fetal development and disease susceptibility. Prenatal exposure to air pollution and heavy metals was associated with altered placental DNA methylation at the global and promoter regions of genes involved in biological processes such as energy metabolism, circadian rhythm, DNA repair, inflammation, cell differentiation, and organ development. The altered placental methylation of these genes was, in some studies, associated with adverse birth outcomes such as low birth weight, small for gestational age, and decreased head circumference. Moreover, few studies indicate that DNA methylation changes in the placenta were sex-specific, and infants born with altered placental DNA methylation patterns were predisposed to developing neurobehavioral abnormalities, cancer, and atopic dermatitis. These findings highlight the importance of more effective and stricter environmental and public health policies to reduce air pollution and protect human health.

## 1. Introduction

Air pollution is an environmental problem that threatens human health and is a major cause of mortality worldwide. In 2019, air pollution caused an estimated 6.67 million deaths, accounting for 12% of all deaths globally [1]. Pregnant women and their developing fetuses are particularly vulnerable to the adverse health effects of air pollution. In pregnant women, the respiratory adaptation to pregnancy leads to an increase in tidal volume and an increase in oxygen consumption [2,3]. Air pollution particles, due to their small size, are inhaled into the lungs, and the smallest particles infiltrate into the bloodstream reaching the placenta and fetus [4,5,6]. Such in utero exposures can affect fetal development, cause adverse birth outcomes, and increase the risk of developing certain diseases in later life, as postulated by the Developmental Origins of Health and Disease (DOHaD) concept [7,8,9,10].

Substantial evidence has associated prenatal air pollution exposure with a range of adverse health outcomes including gestational diabetes [11], preeclampsia [12,13], spontaneous abortions [14,15], preterm births [16,17], low birth weights [18], macrosomia [19], and stillbirths [20]. Furthermore, air pollution exposure during pregnancy was associated with an increased risk of developing cardiovascular diseases [21], neurodevelopmental alterations [22], respiratory problems [23,24], and cancer [25,26]. However, due to the complex composition of air pollution and the intricate processes involved in fetal development, the mechanisms by which air pollution causes these adverse health effects are not yet completely understood.

During the entire pregnancy, the placenta serves as a functional interface that connects the mother to the developing fetus [27]. It secretes hormones and regulates the in utero environment for optimum fetal growth and development. Subsequently, the placenta transfers nutrients from the mother to the fetus and regulates gas and waste exchanges [27]. In this way, the placenta plays a central role in fetal programming, and we suggest that altered placental physiology and function, possibly through epigenetic modifications such as DNA methylation, can provide a mechanism linking prenatal air pollution exposure with pregnancy complications, fetal growth abnormalities, altered newborn phenotypes, and an increased risk of developing certain diseases during the lifespan. This review explores the current knowledge on placental DNA methylation changes associated with prenatal air pollution (including heavy metals) exposure and highlights its effects on fetal development and disease susceptibility.

## 2. Transfer of Air Pollution Particles across the Human Placenta

**Figure 1:**
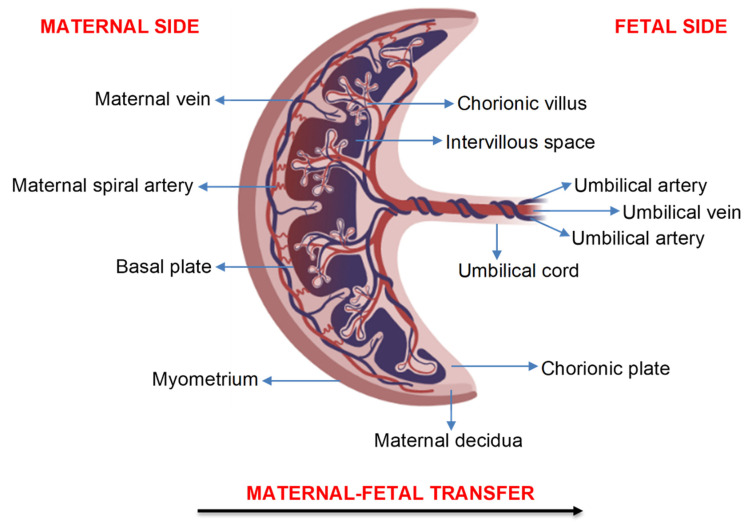
Structure of the human placenta. Created with BioRender.com (accessed on 19 September 2021).

For air pollution particles to directly affect the developing fetus, they must be transported to and/or across the placenta. Figure 1 depicts the structure of the human placenta and indicates the direction of maternal-fetal transfer. Air pollution particles may be transferred to and/or across the placenta via the maternal blood supply through processes such as passive diffusion, active transport, and endocytosis [28]. In a recent review, several studies determined the ability of (ultra)fine particles and nanoparticles to cross the placenta and showed a dependency on the particle’s size, shape, dose, route of exposure, and surface composition [29]. While these studies provide evidence that particles can cross the placenta, most of them were conducted in animal models and did not focus specifically on air pollution particles or heavy metals following real-life exposure conditions.

In the ENVIR*ON*AGE birth cohort, Bové et al. examined the presence of black carbon in placental tissue from 20 healthy, non-smoking women exposed to low (0.63–0.96 µg/m^3^) and high (1.70–2.42 µg/m^3^) levels of residential black carbon during pregnancy [4]. Black carbon particles were detected in all placentae, and the black carbon load was positively correlated with the women’s residential black carbon exposure levels. Furthermore, black carbon particles were found on both the maternal and fetal sides of the placenta, suggesting that black carbon may be transported to the developing fetus [4]. Another study by Liu et al. determined the presence of air pollution nanoparticles in placental tissue cells that were isolated from 15 healthy, non-smoking women exposed to particulate matter with an aerodynamic diameter smaller than 2.5 µm (PM_2.5_: 14.62–18.81 µg/m^3^) and 10 µm (PM_10_: 24.08–31.37 µg/m^3^) during pregnancy [5]. Carbon and metal-containing nanoparticles, usually sourced from heavily trafficked urban roads, were found in macrophage-enriched placental cells, indicating that fine metal particles are phagocytized as foreign bodies in placental tissue [5]. Similarly, Reichrtová et al. investigated the accumulation of two industry and traffic-related air pollutants, lead and nickel, in the placental tissue of 100 women residing in industrial and rural Slovak regions [6]. It was found that both lead and nickel were accumulated in the basal plate, chorionic villi, and chorionic plate of all placentae and that these metals were higher in the placenta from women residing in the industrial region compared to those in the rural region [6]. Since the chorionic villi contain blood vessels that lead directly to the developing fetus, the accumulation of lead and nickel in this region of the placenta implies that these heavy metals can be transported directly to the fetus [6].

## 3. DNA Methylation

Since air pollution particles were shown to translocate into and across the placenta, they may induce placental modifications. Previously, prenatal exposure to air pollution was found to alter placental weight [30], structure [31], and vascular function [32]. This may occur through alterations in placental epigenetic patterns [33,34,35,36,37,38,39,40,41,42,43,44,45,46,47,48,49,50,51,52,53].

Epigenetics is defined as the heritable changes that affect gene expression without altering the DNA nucleotide sequence. The main epigenetic mechanisms are DNA methylation, histone modifications, and microRNAs [54]. Among these, DNA methylation was the most studied epigenetic modification regarding prenatal exposure to air pollution and heavy metals in the placenta, and hence, it was the focus of this review.

**Figure 2:**
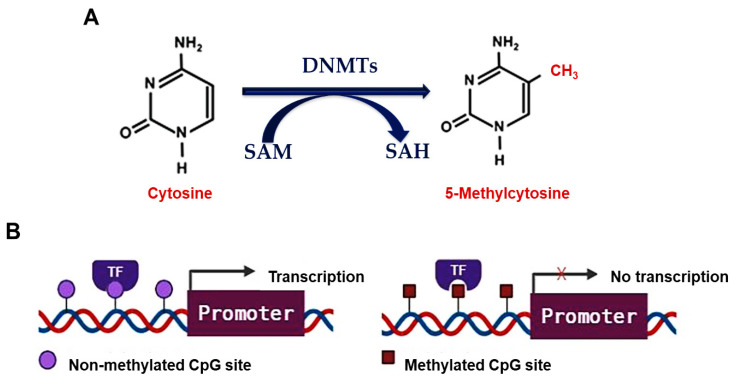
DNA methylation and gene expression regulation. (**A**) Process of DNA methylation. DNMTs covalently add a CH_3_ group from SAM to the number 5 carbon of cytosine bases yielding 5-methylcytosine and SAH. (**B**) Gene expression regulation by DNA methylation. DNA hypomethylation (non-methylated CpG site) enables TF to bind to gene promoters and activate its transcription; however, DNA hypermethylation (methylated CpG site) prevents the binding of TF to gene promoters and inhibits gene transcription. Abbreviations: CH_3_: methyl group; CpG: cytosine bases preceding guanine bases; DNMTs: DNA methyltransferases; SAM: S-adenosylmethionine; SAH: S-adenosylhomocysteine; TF: transcription factor. Created with BioRender.com.

DNA methylation is a biochemical process that occurs predominantly on cytosine bases that precede guanine bases (CpG sites) and involves the covalent addition of a methyl (CH_3_) group to the number 5 carbon of cytosine bases (Figure 2A). This reaction forms 5-methylcytosine and is mediated by DNA methyltransferases (DNMTs), namely DNMT1, DNMT3A, and DNMT3B [55]. DNMT1 maintains cellular methylation levels by recognizing hemi-methylated DNA and preserving the methylation pattern across generations, while DNMT3A and DNMT3B establish new methylation patterns in non-methylated DNA [55]. DNA methylation regulates gene expression by modifying chromatin structure and controlling the accessibility of transcription factors to gene promoter regions (Figure 2B). DNA hypermethylation is often associated with an inactive chromatin structure that prevents transcription factors (TFs) from binding to gene promoters and is involved in gene silencing. In contrast, DNA hypomethylation is associated with an active chromatin structure that enables TFs to bind to gene promoters and initiate transcription [55].

DNA methylation plays a crucial role in fetal development; it creates distinct cell lineages and regulates genome stability, cell proliferation, differentiation, genomic imprinting, and X-chromosome inactivation [54]. During pregnancy, DNA methylation is also involved in fetal epigenetic reprogramming, a process in which DNA methylation marks are erased and re-established, and it is during this time when the fetus is most susceptible to environmental insults [54]. DNA methylation changes in the placenta enable fetal metabolic adaptation in response to environmental stimuli and can affect the cellular phenotype, thus predisposing the fetus to developmental changes with both short-term and long-term health consequences [56].

## 4. Prenatal Air Pollution Exposure and Placental Global DNA Methylation

Numerous studies have shown that prenatal air pollution exposure affects global DNA methylation patterns in the human placenta [33,34,35,36,37,38]. These studies are summarized in Table 1. In the ENVIR*ON*AGE birth cohort, exposure to PM_2.5_ during different stages of pregnancy was associated with a lower degree of placental global DNA methylation, as measured by 5-methyl-deoxycytidine and deoxycytidine levels [33]. In another study, mothers residing near major roadways, an indicator of traffic-related air pollution, showed decreased placental *LINE1* but not *AluYb8* methylation, common markers of global DNA methylation [34]. Furthermore, residing near a major roadway was associated with newborns that had low birth weights; however, the change in placental *LINE1* methylation did not mediate this relationship [34]. In contrast, a Chinese case-control study found that exposure to PM_10_ in the first trimester was associated with decreased *LINE1* methylation in the placenta of fetal growth-restricted newborns [35].

A nested case-control study in Iran observed a positive correlation between global DNA methylation in the placenta (measured as 5-methyl-deoxycytidine and deoxycytidine levels) and exposure to PM_2.5_ and PM_10_ during the first trimester [36]. However, no significant correlation was found between exposures to particulate matter or placental global DNA methylation and birth outcomes such as gestational age, weight, length, and head and chest circumference [36]. The EDEN cohort showed a positive association with PM_10_ exposure the day before birth and placental *Alu* methylation; yet, no significant association was observed with *LINE1* methylation [37]. Furthermore, the EARLI study found that nitrogen dioxide and ozone also induced changes in placental global DNA methylation levels [38]. Together, the above studies showed that prenatal air pollution exposure induced inconsistent DNA methylation changes in the placenta, and there was insufficient evidence linking these air pollution-induced placental global DNA methylation changes with particular birth outcomes or disease susceptibility.

## 5. Prenatal Air Pollution Exposure and Placental Candidate Gene Methylation

Apart from global DNA methylation, prenatal air pollution exposure was found to alter the promoter methylation of placental candidate genes that are involved in key biological processes [34,35,37,38,39,40,41,42,43,44,45]. These studies are summarized in Table 2. In the EDEN cohort, exposure to nitrogen dioxide and PM_10_ altered the methylation patterns of *ADORA2B*, *PXT1*, *KCTD20*, *CAPN10*, *SLC44A5*, *ADCK5*, *TGM6*, *TUBGCP2*, and *KYNU* in placental tissues [37]. These genes function in placental development and were previously associated with hypoxia and preeclampsia [57,58], a pathology that has been linked with air pollution exposure during pregnancy [12,13]. In a separate study, mothers living near major roadways showed differential methylation at seven CpG sites, three of which were located in protein-coding genes (*PTPRN2*, *TMEM125*, and *VPS4A*) [34]. Similarly, in the EARLI cohort, differentially methylated regions were found in five protein-coding genes (*F11R*, *ZNF442*, *SLC25A44*, *STK38*, and *PTPRH*) in the placenta of women exposed to high levels of nitrogen dioxide and ozone during pregnancy [38]. These differentially methylated regions in the placenta did not show DNA methylation changes in cord blood, and hence, they appeared to be tissue-specific. Additionally, differentially methylated regions of three genes (*RNF39*, *CYP2E1*, and *PM20D1*) in cord blood showed consistent nitrogen dioxide and ozone exposure-related altered DNA methylation in the placenta [38], indicating that placental DNA methylation changes may be passed onto the developing fetus. These genes regulate immune and inflammatory responses, and their altered methylation patterns might play a role in preeclampsia.

In China, exposure to PM_10_ during the first and second trimesters increased *HSD11B2* methylation, a gene involved in fetal growth and glucocorticoid metabolism [35]. Furthermore, the increase in *HSD11B2* methylation was more prominent in the placental tissues of fetal growth-restricted newborns compared to the normal-growth newborns. Previously, *HSD11B2* methylation was negatively associated with placental *HSD11B2* gene expression, fetal growth indices, and adverse neurobehavioral outcomes in infants [59]. The Shanghai Mother-Child Pairs Cohort found that exposure to PM_2.5_ during the second and third trimesters, as well as the entire pregnancy, was associated with increased methylation of *BID* and decreased methylation of *IGF2*; these genes are essential for fetal growth [42]. Furthermore, for every 1% increase in *BID* methylation, there was a decrease in head circumference (−1.396 mm, 95% CI: −2.582, −0.209) and biparietal diameter (−0.330 mm, 95% CI: −0.635, −0.026) in the second trimester [42]. In Korea, the COCOA study showed that high PM_2.5_ exposure and low cord blood vitamin D levels during the first trimester were associated with decreased placental methylation of *AHRR*, *DPP10*, and *HLA-DRB1*, and early-onset persistent atopic dermatitis in children [43].

In the ENVIR*ON*AGE birth cohort, PM_2.5_ and black carbon exposure increased placental DNA mutation rates (determined by an increase in the DNA mutation marker, *Alu*) as well as increased the promoter methylation of tumor suppressor (*p53*) and DNA repair (*APEX1*, *OGG1*, and *ERCC4*) genes. This study suggested that prenatal PM_2.5_ and black carbon exposure reduces the DNA repair capacity of the placenta and fetus, which may increase the risk for carcinogenesis in later life [39]. In the same cohort, the analysis of placental tissue from mothers exposed to PM_2.5_ in the first and third trimesters showed altered methylation of the genes (*CLOCK*, *CRY1*, *NPAS2*, and *PER1–3*) involved in circadian rhythm regulation [40]. Previously, dysregulation in placental methylation of these circadian pathway genes was associated with preeclampsia [60]. Another study indicated that second-trimester PM_2.5_ exposure decreased promoter methylation of the *Lep* gene, a hormone involved in intrauterine development, embryo implantation, energy regulation, and fetal growth [41]. Interestingly, the promoter methylation of *Lep* was higher in the placental tissue of male neonates compared to those of female neonates, suggesting that altered DNA methylation in the placenta may be sex-specific [41].

Prenatal air pollution exposure was also associated with altered placental mitochondrial DNA methylation [44,45]. In the ENVIR*ON*AGE cohort, placental mitochondrial DNA methylation was analyzed in the *D-loop* control region and *MT-RNR1* region. First trimester PM_2.5_ exposure was associated with increased mitochondrial DNA methylation at both the *D-loop* and *MT-RNR1* regions [44]. An increase in *D-loop* (non-significant) and *MT-RNR1* (significant) methylation was also observed in placental tissue following exposure to PM_2.5_ for the entire pregnancy [44]. These findings were confirmed in a smaller sample population of the ENVIR*ON*AGE cohort, where PM_2.5_ and black carbon exposure, throughout pregnancy, non-significantly increased *D-loop* and *LDLR* methylation [45]. In both studies, mitochondrial DNA methylation was negatively associated with mitochondrial DNA content (a measure of damaged mitochondria and mitophagy) [44,45]. PM_2.5_ and black carbon exposure for the entire pregnancy also decreased placental promoter methylation of *PINK1*, a gene involved in mitochondrial quality control and mitophagy [45]. Moreover, a 0.42% increase in *D-loop* methylation was associated with decreased newborn birth weight (−106.98 g, 95% CI: −209.60 g, −4.36 g) [45].

## 6. Prenatal Heavy Metal Exposure and Placental DNA Methylation

Heavy metals such as arsenic, cadmium, lead, manganese, mercury, and nickel are common constituents of industry and traffic-related air pollution. Particulate matter, mainly PM_2.5_ and PM_10_, has a strong potential for adsorbing heavy metals, which then enter the human body through inhalation [61]. There is increasing evidence that heavy metals bound to particulate matter play a crucial role in the adverse health effects caused by particulate matter [62,63,64]. Therefore, we also included studies investigating placental DNA methylation changes and prenatal exposure to heavy metals [46,47,48,49,50,51,52,53]. These studies are summarized in Table 3.

Most studies on prenatal heavy metal exposure and placental DNA methylation changes were conducted within the RICHS cohort. In the RICHS cohort, maternal exposure to high levels of arsenic, cadmium, lead, mercury, and manganese were associated with increased placental *NR3C1* methylation compared to the low exposure groups [46]. Placental *NR3C1* plays a vital role in cognitive and neurodevelopment by regulating the development of the child’s hypothalamic-pituitary-adrenal (HPA) axis and cortisol levels. Therefore, altered placental *NR3C1* methylation may provide insight into cognitive and neurodevelopmental abnormalities in children in later life [46]. In a separate study, mothers with high toenail cadmium concentrations were found to have low levels of placental *PCDHAC1* methylation and were at an increased odds of giving birth to an infant that was small for gestational age or with a decreased head circumference [50]. Another study showed that high placental cadmium levels were associated with differential methylation at 17 CpG sites, and DNA methylation at 9 of these 17 CpG sites was associated with increased expression of genes involved in inflammatory signaling and cell growth (*TNFAIP2*, *EXOC3L4*, *GAS7*, *SREBF1*, *ACOT7*, and *RORA*) [51]. Furthermore, high placental expressions of *TNFAIP2* and *ACOT7* were associated with decreased birth weight (Tau: −0.099, *p* = 0.039 and Tau: −0.134, *p* = 0.0048, respectively). High placental expression of *ACOT7* was also associated with decreased birth length (Tau: –0.106, *p* = 0.029) and decreased head circumference (Tau: −0.145, *p* = 0.0032) [51]. Moreover, within the RICHS cohort, exposure to mercury, measured in infant toenails, was associated with differential methylation at 339 loci; 10 of these differentially methylated loci resided in the *CPLX1*, *TTC23*, and *EMID2* genes and were associated with a high risk for adverse neurobehavioral profiles [52]. Exposure to high levels of manganese, measured in infant toenails, was associated with differential methylation at 5 CpG loci (*EMX2OS*, *ATAD2B*, *FTO/RPGRIP1L*, *EN1*, and *LOC284276*). These CpG loci resided in genes involved in neurodevelopment (*EMX2OS*, *ATAD2B*, and *EN1*) and fetal growth (*FTO/RPGRIP1L*) [53]. The function of *LOC284276* is currently unknown; however, for every 10% increase in placental *LOC284276* methylation, there was a decrease in birth weight (−293.44 g, *p* = 0.018) [53].

In the New Hampshire Birth Cohort Study, placental arsenic levels were associated with differential methylation at 163 CpG sites. Of these, 13 CpG sites attained genome-wide significance and were located at the *LYRM2* (11 CpG sites), *CAMTA1* (1 CpG site), and *CCDC57* (1 CpG site) genes [48]. A nested cohort in Bangladesh found that maternal exposure to arsenic, via contaminated drinking water, was associated with hypermethylation at several CpG sites, which were mainly located within open sea regions [47]. Moreover, prenatal arsenic exposure was associated with CpG methylation at the *NR3C1* gene (unadjusted analysis) and the *TRA2B*, *PLCE1*, and *CD36* genes (adjusted analysis) [47].

The omega cohort and pilot case-control placenta microarray study showed that cadmium levels were higher in the placental tissues from female neonates (5 ng/g) compared to male neonates (2 ng/g). High cadmium levels were associated with hypomethylation at three CpG sites (*ARL9*, *SIAH3*, and *HS3ST4*) and one genomic region (region 86974674 to 86975244 on chromosome 7; *CROT* and *TP53TG1*) in the placental tissue of female neonates [49]. In the placental tissue of male neonates, high cadmium levels were associated with hypomethylation at two CpG sites (*MECOM*) and two genomic regions (region 169379554 to 169380078 on chromosome 3 and region 1792758 to 1792758 on chromosome 8; *MECOM* and *ARHGEF10*) as well as hypermethylation at one CpG site (*SALL1*) [49]. These differentially methylated genes are involved in cell damage response (*SIAH3*, *HS3ST4*, and *TP53TG1*) in females and cell differentiation, angiogenesis, and organ development (*MECOM* and *ARHGEF10*) in males. These results suggest that cadmium-associated placental DNA methylation changes may induce fetal growth abnormalities in a sex-dependent manner [49].

## 7. Conclusions

As a natural barrier that directly connects the mother to the developing fetus, the placenta is continuously in contact with substances to which both the mother and fetus are exposed [27]. Perturbations in the maternal environment can be transferred to the fetus through altered placental functions, a concept known as fetal reprogramming. As a result, the placenta, which is genetically identical to the fetus, contains important information on the in utero fetal life and can be considered as a “mirror” of the future health and development of the newborn [40]. The studies included in this review provide evidence that air pollution particles, including heavy metals, can be transported to the placenta [4,5,6]. Moreover, the finding of air pollution particles and heavy metals on both the maternal and fetal sides of the placenta suggests a strong possibility that these particles may be transported directly to the developing fetus. Air pollution particles and heavy metals in the placenta can modify placental functions by altering the DNA methylome, which mediates the transcriptional activation and silencing of genes involved in various physiological and developmental processes [33,34,35,36,37,38,39,40,41,42,43,44,45,46,47,48,49,50,51,52,53]. The effect of air pollution exposure on DNA methylation changes during the first 1000 days of life has been systematically reviewed [65]; however, this review did not include heavy metals as a constituent of air pollution.

**Figure 3:**
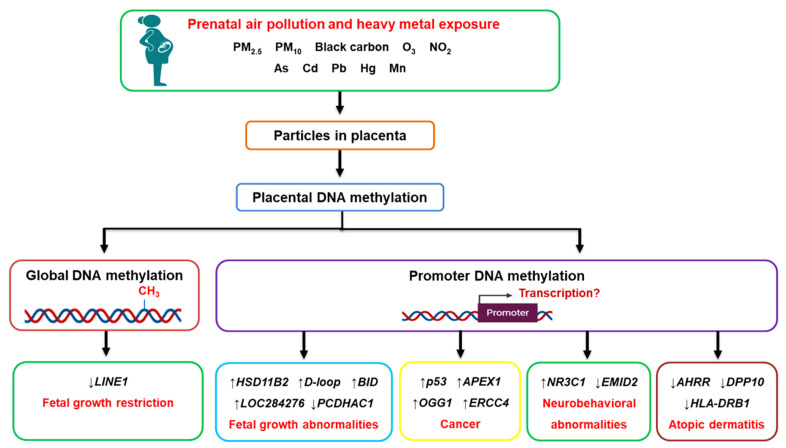
The effects of prenatal air pollution exposure on placental DNA methylation patterns and its implications on fetal development and future disease susceptibility. Maternal exposure to air pollution, including heavy metals, can reach the placenta, where they alter DNA methylation patterns at both the global and gene promoter level. The aberrant methylation of genes affects fetal growth (*HSD11B2*, *BID*, *D-loop*, *PCDHAC1*, *LOC284276*) and increases the risk of developing cancer (*p53*, *APEX1*, *OGG1*, *ERCC4*), neurobehavioral abnormalities (*NR3C1*, *EMID2*), and atopic dermatitis (*AHRR*, *DPP10*, *HLA-DRB1*) in later life. Abbreviations: AHRR: aryl hydrocarbon receptor repressor; APEX1: AP endonuclease 1; As: arsenic; BID: BH3 interacting domain death agonist; Cd: cadmium; D-loop: displacement loop control region (heavy strand); DPP10: dipeptidyl peptidase 10; EMID2: EMI domain-containing protein 2; ERCC4: excision repair 4; Hg: mercury; HLA-DRB1: HLA class II histocompatibility antigen, DRB1 beta chain; HSD11B2: 11β-hydroxysteroid dehydrogenase 2; LINE1: long interspersed nuclear element 1; Mn: manganese; NO_2_: nitrogen dioxide; NR3C1: nuclear receptor subfamily 3 group C member 1 glucocorticoid receptor; O_3_: ozone; OGG1: oxoguanine glycosylase 1; p53: tumor suppressor protein 53; Pb: lead; PCDHAC1: protocadherin alpha subfamily C1; PM_2.5_: particulate matter with an aerodynamic diameter smaller than 2.5 µm; PM_10_: particulate matter with an aerodynamic diameter smaller than 10 µm.

Prenatal exposure to air pollution and heavy metals was associated with altered placental DNA methylation at both the global and candidate gene promoter levels [33,34,35,36,37,38,39,40,41,42,43,44,45,46,47,48,49,50,51,52,53] (summarized in Figure 3); however, whether the changes in gene promoter methylation affected the expression of the gene was not established in the majority of studies. Aside from the studies on placental mitochondrial DNA methylation [44,45], no genes were investigated in more than one study. This makes it difficult to determine if the alterations in candidate gene promoter methylation are specific to a particular socio-demographic population or to prenatal air pollution exposure in a certain geographical location. Different locations have different sources, and chemical compositions of air pollution and individual components are not often encountered in isolation within natural settings.

Two studies also showed that PM_2.5_ and cadmium-induced DNA methylation of genes were different in the placentae from male and female neonates [41,49], suggesting that the adverse health effects of these air pollutants may be sex-specific. Furthermore, prenatal exposure to air pollution induced inconsistent placental global DNA methylation changes, which may be the result of several other factors such as maternal diet or nutritional status and folate supplementation; folate is an essential micronutrient and methyl donor required during pregnancy to prevent neural tube defects in the fetus. Interestingly, trimester-specific analyses indicated that most of the placental DNA methylation changes observed at birth followed air pollution exposures during the early stages of pregnancy [33,35,36,37,40,43,44], which is also when fetal epigenetic reprogramming occurs. Although this suggests that air pollution and heavy metal exposure during the early stages of pregnancy may, through altered placental DNA methylation, be responsible for fetal growth abnormalities, there was insufficient evidence linking prenatal air pollution and heavy metal-induced placental DNA methylation changes with specific diseases. Follow-up studies are required to determine if placental DNA methylation changes persist to the fetus and into adulthood and its possible implications on fetal development and disease susceptibility throughout the lifespan. Together, the findings depicted in this review highlight the need for more effective and stricter environmental and public health policies to reduce air pollution and protect human health.

## Figures and Tables

**Table 1:** Studies on prenatal air pollution exposure and placental global DNA methylation.

Author	Study	Sample Size	Method	Air Pollutant	Duration Exposed	Findings
Janssen et al. [33]	ENVIR*ON*AGE cohort, Belgium	240	UPLC/MS-MS	PM_2.5_: 5 µg/m^3^ increment	Implantation (6–21 days after conception)	↓ Global DNA methylation (−1.08%, 95% CI: −1.80, −0.36%, *p* = 0.004)
				PM_2.5_: 5 µg/m^3^ increment	First trimester	↓ Global DNA methylation (−2.41%, 95% CI: −3.62, −1.20%, *p* = 0.0001)
				PM_2.5_: 5 µg/m^3^ increment	Second trimester	↓ Global DNA methylation (−1.51%, 95% CI: −2.66, −0.36%, *p* = 0.01)
				PM_2.5_: 5 µg/m^3^ increment	Entire pregnancy	↓ Global DNA methylation (−2.19%, 95% CI: −3.65, −0.73%, *p* = 0.004)
Kingsley et al. [34]	RICHS cohort, US	471	Bisulfite-PCR pyrosequencing	Traffic-related air pollution: Women residing ≤ 150 m from a major roadway or ≤50 m from a secondary road	Entire pregnancy	Residing near a major roadway: ↓ *LINE1* methylation (−0.82%, 95% CI: −1.57, −0.07%, *p* = 0.03); No significant association with *AluYb8* methylation (*p* = 0.07)
Cai et al. [35]	Case-control study, China	181	Bisulfite-PCR pyrosequencing	PM_10_: 10 µg/m^3^ increment	First trimester	Placenta of fetal growth restricted newborns: ↓ *LINE1* methylation (−1.78%, 95% CI: −3.35, −0.22%, *p* < 0.05) Placenta of normal growth newborns: No significant association with *LINE1* methylation (*p* > 0.05)
Maghbooli et al. [36]	Nested case-control study, Iran	92	RP-HPLC	PM_2.5_: 20.43 ± 0.68 µg/m^3^ and 30.99 ± 0.86 µg/m^3^ PM_10_: 64.97 ± 2.52 µg/m^3^ and 74.34 ± 2.66 µg/m^3^	First trimester	PM_2.5_: ↑ Global DNA methylation (*r* = 0.26, *p* = 0.01) PM_10_: ↑ Global DNA methylation (*r* = 0.38, *p* = 0.0001)
Abraham et al. [37]	EDEN cohort, France	668	Illumina Infinium HumanMethylation450K BeadChip	PM_10_: 10 µg/m^3^ increment	Day before birth	↑ *Alu* methylation (*β* = 0.08, *p* = 0.01); No significant association with *LINE1* methylation (*β* = 0.09, *p* = 0.28)
Ladd-Acosta et al. [38]	EARLI cohort, US	124	Illumina Infinium HumanMethylation450K BeadChip	NO_2_ and O_3_	Entire pregnancy	O_3_: ↓ DNA methylation at shelf regions (*p* = 0.00028), ↑ DNA methylation at CpG islands (*p* = 0.00295) and shore regions (*p* = 0.00266) NO_2_: ↓ DNA methylation in CpG islands (*p* = 0.00359) and shore regions (*p* = 0.04284)

↓: decrease; ↑: increase; Alu/AluYb8: arthrobacter luteus elements; CI: confidence interval; EARLI: Early Autism Risk Longitudinal Investigation; EDEN: Etude de cohorte g’en’eraliste men’ee en France sur les D’eterminants pr’e et post natals pr’ecoces du d´eveloppement psychomoteur et de la sant´e de l’Enfant; ENVIR*ON*AGE: ENVIRonmental influence *ON* early AGEing; LINE1: long interspersed nuclear element 1; MS-MS: tandem mass spectrometry; NO_2_: nitrogen dioxide; O_3_: ozone; PM_2.5_: particulate matter with an aerodynamic diameter smaller than 2.5 µm; PM_10_: particulate matter with an aerodynamic diameter smaller than 10 µm; RICHS: Rhode Island Child Health Study; RP-HPLC: reversed-phase high-pressure liquid chromatography; UPLC: ultra-pressure liquid chromatography; US: United States.

**Table 2:** Studies on prenatal air pollution exposure and placental candidate gene methylation.

Author	Study	Sample Size	Technique	Air Pollutant	Duration Exposed	Findings
Kingsley et al. [34]	RICHS cohort, US	215	Illumina Infinium HumanMethylation450K BeadChip	Traffic-related air pollution: Women residing ≤150 m from a major roadway or ≤50 m from a secondary road	Entire Pregnancy	Residing near a major roadway: Differential methylation of 7 CpG sites—4 were mapped to non-genic regions and 3 were mapped to genes. ↑ *PTPRN2* methylation (+0.061%, *p* = 2.904 × 10^−6^), ↓ *TMEM125* methylation (−0.012%, *p* = 1.077 × 10^−3^), ↓ *VPS4A* methylation (−0.016%, *p* = 3.151 × 10^−5^)
Cai et al. [35]	Case-control study, China	181	Bisulfite-PCR pyrosequencing	PM_10_: 10 µg/m^3^ increment	First trimester	Placenta of fetal growth restricted newborns: ↑ *HSD11B2* methylation (+1.03%, 95% CI: 0.07, 1.98%, *p* < 0.05) Placenta of normal growth newborns: No significant association with *HSD11B2* methylation (*p* > 0.05)
				PM_10_: 10 µg/m^3^ increment	Second trimester	Placenta of fetal growth restricted newborns: ↑ *HSD11B2* methylation (+2.23%, 95% CI: 0.69, 3.76%, *p* < 0.05) Placenta of normal growth newborns: No significant association with *HSD11B2* methylation (*p* > 0.05) Total population: ↑ *HSD11B2* methylation (+1.42%, 95% CI: 0.24, 2.57%, *p* < 0.05)
				PM_10_: 10 µg/m^3^ increment	Entire pregnancy	Total population: ↑ *HSD11B2* methylation (+1.98%, 95% CI: 0.53, 3.43%, *p* < 0.05)
Abraham et al. [37]	EDEN cohort, France	668	Illumina Infinium HumanMethylation450K BeadChip	NO_2_: 10 µg/m^3^ increment	First trimester	↓ *ADORA2B* methylation at 2 CpG sites (cg17580614: *β* = −0.037, *p* < 0.001; cg07563400: *β* = −0.042, *p* < 0.001)
				NO_2_: 10 µg/m^3^ increment	Second trimester	↓ *ADORA2B* methylation at 2 CpG sites (cg17580614: *β* = −0.044, *p* < 0.0001; cg07563400: *β* = −0.047, *p* < 0.0001), ↑ *PXT1/KCTD20* methylation (cg10984505: *β* = 0.002, *p* = 0.02
				NO_2_: 10 µg/m^3^ increment	Third trimester	↓ *CAPN10* methylation (cg01712700: *β* = −0.004, *p* = 0.02)
				PM_10_: 10 µg/m^3^ increment	One month before birth	↑ *SLC44A5* methylation (cg12659128: *β* = 0.037, *p* = 0.03), ↑ *ADCK5* methylation (cg23075260: *β* = 0.018, *p* = 0.03), ↑ *TGM6* methylation (cg06967014: *β* = 0.007, *p* = 0.03), ↓ *TUBGCP2* methylation (cg05142592: *β* = −0.008, *p* = 0.03)
				PM_10_: 10 µg/m^3^ increment	3 days before birth	↓ *KYNU* methylation (cg04112100: *β* = −0.012, *p* = 0.04)
Ladd-Acosta et al. [38]	EARLI cohort, US	124	Illumina Infinium HumanMethylation450K BeadChip	NO_2_ and O_3_	Entire pregnancy	Differentially methylated regions in 5 genes that seemed to be specific to placental tissue: *ZNF442*, *PTPRH*, *SLC25A44*, *F11R*, and *STK38* Differentially methylated regions in 3 genes found in cord blood and showed similar methylation patterns in placental tissue: *RNF39*, *CYP2E1*, and *PM20D1*
Neven et al. [39]	ENVIRONAGE cohort, Belgium	463	Bisulfite-PCR pyrosequencing	PM_2.5_: 3.84 µg/m^3^ increment	Entire pregnancy	↑ *APEX1* methylation (+7.34%, 95% CI: 0.52, 14.16%, *p* = 0.0089), ↑ *OGG1* methylation (+13.06%, 95% CI: 3.88, 22.24%, *p* = 0.0054), ↑ *ERCC4* methylation (+ 16.31%, 95% CI: 5.43, 27.18%, *p* = 0.0034), ↑ *p53* methylation (+10.60%, 95% CI: 4.46, 16.74%, *p* = 0.0008), ↓ *DAPK1* methylation (−12.92%, 95% CI: −22.35, −3.49%, *p* = 0.0073)
				Black carbon: 0.36 µg/m^3^ increment	Entire pregnancy	↑ *APEX1* methylation (+9.16%, 95% CI: 4.06, 14.25%, *p* = 0.005), ↑ *ERCC4* methylation (+27.56%, 95% CI: 17.58, 37.55%, *p* < 0.0001)
Nawrot et al. [40]	ENVIRONAGE cohort, Belgium	407	Bisulfite-PCR pyrosequencing	PM_2.5_: 7.9 µg/m^3^ increment	First trimester	↓ *CLOCK* methylation (−0.59 Log (fold-change), 95% CI: −0.93, −0.25, *p* = 0.0007)
				PM_2.5_: 8.9 µg/m^3^ increment	Third trimester	↑ *NPAS2* methylation (+0.16 Log (fold-change), 95% CI: 0.06, 0.27, *p* = 0.002), ↑ *CRY1* methylation (+0.59 Log (fold-change), 95% CI: 0.22, 0.95, *p* = 0.002), ↑ *PER2* methylation (+0.36 Log (fold-change), 95% CI: 0.16, 0.57, *p* = 0.0005), ↑ *PER3* methylation (+0.42 Log (fold-change), 95% CI: 0.18, 0.67, *p* = 0.0008), ↓ *PER1* methylation (−0.51 Log (fold-change), 95% CI: −0.90, −0.13, *p* = 0.01)
				PM_2.5_: 9.7 µg/m^3^ increment	Last month of pregnancy	↑ *CRY1* methylation (*p* = 0.01), ↑ *PER2* methylation (*p* = 0.0003), ↑ *PER3* methylation (*p* = 0.02)
Saenen et al. [41]	ENVIRONAGE cohort, Belgium	361	Bisulfite-PCR pyrosequencing	PM_2.5_: 7.5 µg/m^3^ increment	Second trimester	↓ *Lep* methylation (−1.4%, 95% CI: −2.7, −0.19%, *p* = 0.02), ↑ *Lep* methylation in placentae from male neonates compared to placentae from female neonates (+1.33%, 95% CI: 0.40, 2.27%, *p* = 0.005)
Zhao et al. [42]	Shanghai MCPC, China	287	Bisulfite-PCR pyrosequencing	PM_2.5_: 1 µg/m^3^ increment	Second trimester	↓ *IGF2* methylation (−0.135%, 95% CI: −0.236, −0.034), ↑ *BID* methylation (+ 0.132%, 95% CI: 0.047, 0.217), ↑ *FOXN3* methylation (position 1, +0.091, 95% CI: 0.008, 0.174)
				PM_2.5_: 1 µg/m^3^ increment	Third trimester	↓ *IGF2* methylation (−0.229%, 95% CI: −0.384, −0.073), ↑ *BID* methylation (+ 0.209%, 95% CI: 0.072, 0.346)
				PM_2.5_: 1 µg/m^3^ increment	Entire pregnancy	↓ *IGF2* methylation (−0.297%, 95% CI: −0.489, −0.105), ↑ *BID* methylation (+ 0.209%, 95% CI: 0.039, 0.380)
Yang et al. [43]	COCOA study, Korea	1180	Illumina Infinium HumanMethylationEPIC BeadChip	PM_2.5_	First trimester (3–7 weeks of pregnancy)	Placenta of children with high PM_2.5_ exposure, low cord blood vitamin D levels, and atopic dermatitis: ↓ *AHRR* methylation (cg16371648: *β* = −0.367, *p* = 0.026), ↓ *DPP10* methylation (cg19211931: *β* = −0.263, *p* = 0.013), ↓ *HLA-DRB1* methylation (cg10632894: *β* = −0.318, *p* = 0.026)
Janssen et al. [44]	ENVIRONAGE cohort, Belgium	381	Bisulfite-PCR pyrosequencing	PM_2.5_: 7.8 µg/m^3^ increment	First trimester	↑ *D-loop* methylation (+0.44%, 95% CI: 0.12, 0.75%, *p* < 0.05), ↑ *MT-RNR1* methylation (+1.27%, 95% CI: 0.23, 2.32%, *p* < 0.05)
				PM_2.5_: 3.0 µg/m^3^ increment	Entire pregnancy	↑ *D-loop* methylation (+0.21%, 95% CI: -0.003, 1.02%, *p* > 0.05), ↑ *MT-RNR1* methylation (+0.91, 95% CI: 0.56, 4.18%, *p* < 0.05)
Vos et al. [45]	ENVIRONAGE cohort, Belgium	60	Bisulfite-PCR pyrosequencing	PM_2.5_ (5.4 µg/m^3^ increment) and black carbon (0.9 µg/m^3^ increment)	Entire pregnancy	↑ *D-loop* methylation (+0.47%, 95% CI: 0.20, 0.73%, *p* = 0.61), ↑ *LDLR2* methylation (+0.81%, 95% CI: −0.17, 1.78, *p* = 0.09), ↓ *PINK1* methylation (−0.42%, 95% CI: −0.60, −0.24%, *p* < 0.05)

↑: increase; ↓: decrease; ADCK5: aarF domain containing kinase; ADORA2B: adenosine A2B receptor; AHRR: aryl hydrocarbon receptor repressor; APEX1: AP endonuclease 1; BID: BH3 interacting domain death agonist; CAPN10: calcium-activated neutral proteinase 10; CI: confidence interval; CLOCK: circadian locomotor output cycles kaput; COCOA: Cohort for Childhood Origin of Asthma and Allergic Diseases; CpG: cytosine bases preceding guanine bases; CRY1: cryptochrome circadian clock 1; CYP2E1: cytochrome P450 2E1; DAPK1: death-associated protein kinase 1; D-loop: displacement loop control region (heavy strand); DPP10: dipeptidyl peptidase 10; EARLI: Early Autism Risk Longitudinal Investigation; EDEN: Etude de cohorte g´en´eraliste men´ee en France sur les D´eterminants pr´e et post natals pr´ecoces du d´eveloppement psychomoteur et de la sant´e de l’Enfant; ENVIR*ON*AGE: ENVIRonmental influence *ON* early AGEing; ERCC4: excision repair 4; F11R: F11 receptor; FOXN3: forkhead box N3; HLA-DRB1: HLA class II histocompatibility antigen, DRB1 beta chain; HSD11B2: 11β-hydroxysteroid dehydrogenase 2; IGF2: insulin-like growth factor 2; KCTD20: potassium channel tetramerization domain containing 20; KYNU: kynureninase; LDLR2: displacement loop control region (light strand); Lep: leptin; MCPC: Mother Child Pairs Cohort; MT-RNR1: mitochondrial region RNR1; NO_2_: nitrogen dioxide; NPAS2: neuronal PAS domain-containing protein 2; O_3_: ozone; OGG1: oxoguanine glycosylase 1; p53: tumor suppressor protein 53; PER1-3: period circadian clock 1, 2, or 3; PINK1: PTEN-induced kinase 1; PM_2.5_: particulate matter with an aerodynamic diameter smaller than 2.5 µm; PM_10_: particulate matter with an aerodynamic diameter smaller than 10 µm; PM20D1: Peptidase M20 domain containing 1; PTPRH: protein tyrosine phosphatase receptor type H; PTPRN2: protein tyrosine phosphatase receptor type N2; PXT1: peroxisomal testis specific 1; RICHS: Rhode Island Child Health Study; RNF39: ring finger protein 39; SLC25A44: solute carrier family 25 member 44; SLC44A5: solute carrier family 44 member 5; STK38: serine/threonine kinase 38; TGM6: transglutaminase 6; TMEM125: transmembrane protein 125; TUBGCP2: tubulin gamma complex associated protein 2; US: United States; VPS4A: vacuolar protein sorting 4 homolog A; ZNF442: zinc finger protein 442.

**Table 3:** Studies on prenatal heavy metal exposure and placental DNA methylation.

Author	Study	Sample Size	Method	Heavy Metal	Findings
Appleton et al. [46]	RICHS cohort, US	222	Bisulfite-PCR pyrosequencing	Arsenic (0.14 µg/g; measured in toenail clippings)	↑ *NR3C1* methylation (+0.71, *p* = 0.0002)
				Cadmium (0.17 µg/g; measured in toenail clippings)	↑ *NR3C1* methylation (+0.74, *p* < 0.001)
				Lead (2.3 µg/g; measured in toenail clippings)	↑ *NR3C1* methylation (+0.77, *p* = 0.004)
				Mercury (0.17 µg/g; measured in toenail clippings)	↑ *NR3C1* methylation (+1.41, *p* < 0.001)
				Manganese (2.2 µg/g; measured in toenail clippings)	↑ *NR3C1* methylation (+0.80, *p* = 0.02)
Cardenas et al. [47]	Nested cohort, Bangladesh	37	Illumina Infinium HumanMethylation450 BeadChip	Arsenic (63.7 ± 116.5 µg/L; measured in maternal drinking water by ICP-MS)	CpG methylation at 3 genes—*TRA2B*, *PLCE1*, and *CD36*; hypermethylation of open sea regions
Green et al. [48]	NHBCS, US	285	Illumina Infinium HumanMethylation450K BeadChip	Arsenic (0.82 µg/kg; measured in placental tissue by ICP-MS)	Differential methylation at 163 CpG sites (*q* < 0.05). Of these, 13 attained genome-wide significance and were tracked to *LYRM2* (11 CpG sites), *CAMTA1* (1 CpG site), and *CCDC57* (1 CpG site) genes
Mohanty et al. [49]	Omega cohort, Pacific Northwest Placenta MicroArray Study (pilot case-control study)	24	Illumina Infinium HumanMethylation450K BeadChip	Cadmium (5 ng/g in placental tissue from female neonates and 2 ng/g in placental tissue from male neonates; measured by ICP-MS)	Placenta of female neonates: hypomethylation of 3 CpG sites located near *ARL9* (*p* = 0.01), *SIAH3* (*p* = 0.08), and *HS3ST4* (*p* = 0.08) genes; hypomethylation of 1 genomic region on chromosome 7 (region 86974674 to 86975244, including *CROT* and *TP53TG1* genes; *p* = 0.06) Placenta of male neonates: hypomethylation of 2 CpG sites located near *MECOM* (*p* < 0.01); hypermethylation of 1 CpG site located near *SALL1* (*p* = 0.08); hypomethylation of 2 genomic regions (region 169379554 to 169380078 on chromosome 3, including the *MECOM* gene (*p* = 0.03) and region 1792758 to 1792758 on chromosome 8, including the *ARHGEF10* gene (*p* = 0.07))
Everson et al. [50]	RICHS cohort, US	94	Illumina Infinium HumanMethylation450K BeadArray	Cadmium (0.01 µg/g; measured in maternal toenail clippings by ICP-MS)	↓ *PCDHAC1* methylation (TSS200 and TSS1500)
Everson et al. [51]	NHBCS and RICHS cohort, US	343 (NHBCS) 141 (RICHS cohort)	Illumina Infinium HumanMethylation450K BeadArray	Cadmium (3.13 ng/g (NHBCS) and 4.37 ng/g (RICHS cohort); measured in placental tissue by ICP-MS)	Differential methylation of 17 CpG sites (*p* < 1 × 10^−5^); DNA methylation at 9 of these 17 CpG sites were associated with ↑ expression of *TNFAIP2*, *EXOC3L4*, *GAS7*, *SREBF1*, *ACOT7*, and *RORA*
Maccani et al. [52]	RICHS, US	41	Illumina Infinium HumanMethylation450 BeadArray	Mercury (0.077–0.425 μg/g; measured in infant toenail clippings)	Differential methylation at 339 loci; 10 loci residing in *CPLX1*, *TTC23*, and *EMID2* were associated with a high risk for adverse neurobehavioral profiles (*p* < 0.01)
Maccani et al. [53]	RICHS cohort, US	61	Illumina Infinium HumanMethylation450 BeadChip	Manganese (0.858–5.666 μg/g; measured in infant toenail clippings)	Differential methylation at 5 CpG loci: *EMX2OS* (cg16063747; *p* = 3.15 × 10^−8^), *ATAD2B* (cg08192560; *p* = 3.48 × 10^−8^), *FTO/RPGRIP1L* (cg26692097; *p* = 8.69 × 10^−8^), *EN1* (cg07419575; *p* = 1.26 × 10^−7^), and *LOC284276* (cg22284422; *p* = 1.29 × 10^−7^)

↑: increase; ↓: decrease; ACOT7: acyl-CoA thioesterase 7; ARL9: ADP-ribosylation factor-like 9; ARHGEF10: rho guanine nucleotide exchange factor 10; ATAD2B: ATPase family AAA domain containing 2B; CAMTA1: calmodulin-binding transcription activator-1; CCDC57: coiled-coil domain containing 57; CD36: cluster of differentiation 36; CpG: cytosine bases preceding guanine bases; CPLX1: complexin 1; CROT: carnitine O-octanoyltransferase; EMID2: EMI domain containing protein 2; EMX2OS: EMX2 opposite strand; EN1: engrailed 1 (homeobox protein); EXOC3L4: exocyst complex component 3 like 4; FTO: fat mass and obesity-associated protein; GAS7: growth arrest specific 7; HS3ST4: heparin sulfate (glucosamine) 3-O-sulfotransferase 4; ICP-MS: inductively coupled plasma mass spectrometry; LYRM2: LYR-motif containing 2; MECOM: MDS1 and EVI1 complex locus; NHBCS: New Hampshire Birth Cohort Study; NR3C1: nuclear receptor subfamily 3 group C member 1 glucocorticoid receptor; PCDHAC1: protocadherin alpha subfamily C1; PLCE1: phospholipase C epsilon 1; RICHS: Rhode Island Child Health Study; RORA: retinoic acid receptor-related orphan receptor alpha; RPGRIP1L: retinitis pigmentosa GTPase regulator-interacting protein 1 like; SALL1: spalt-like transcription factor 1; SIAH3: siah E3 ubiquitin protein ligase family member 3; SREBF1: sterol regulatory element binding factor 1; TNFAIP2: tumor necrosis factor alpha induced protein 2; TP53TG1: TP53 target 1; TRA2B: transformer 2 beta homolog; TTC23: tetratricopeptide repeat domain 23; US: United States.
